# The Genetic Origin of Short Tail in Endangered Korean Dog, DongGyeongi

**DOI:** 10.1038/s41598-017-10106-6

**Published:** 2017-08-30

**Authors:** DongAhn Yoo, Kwondo Kim, Hyaekang Kim, Seoae Cho, Jin Nam Kim, Dajeong Lim, Seog-Gyu Choi, Bong-Hwan Choi, Heebal Kim

**Affiliations:** 1 C&K genomics, C-1008, H businesspark, 26, Beobwon-ro 9-gil, Songpa-gu, Seoul, Republic of Korea; 2 Interdisciplinary Program in Bioinformatics, Seoul National University, Seoul, Republic of Korea; 3 Department of Agricultural Biotechnology and Research Institute of Agriculture and Life Sciences, Seoul National University, Seoul, Republic of Korea; 4 Institute of Conservation Gyeongju Donggyeong Dog, Dongguk University, Gyeongju, 780-714 Republic of Korea; 5 National Institute of Animal Science, RDA, Wanju, 565-851 Republic of Korea

## Abstract

The tail of many animal species is responsible for various physiological functions. The functional importance of tail may have brought tail-loss to attention in many evolutionary and developmental studies. To provide a better explanation for the loss of tail, the current study aims to identify the evolutionary history and putative causal variants for the short tail in DongGyeongi (DG), an endangered dog breed, which is also the only dog in Korea that possesses a short tail. Whole genome sequencing was conducted on 22 samples of DG, followed by an investigation of population stratification with 10 other dog breeds. The genotypes, selective sweep and demography of DG were also investigated. As a result, we discovered the unique genetic structure of DG and suggested two possible ways in which the short tail phenotype developed. Moreover, this study suggested that selective sweep genes, ANKRD11 and ACVR2B may contribute to the reduction in tail length, and non-synonymous variant in the coding sequence of T gene and the CpG island variant of SFRP2 gene are the candidate causal variants for the tail-loss.

## Introduction

Tail is one of the common phenotypes found in many animal species^1^. As a physical structure that is conserved in many organisms, it serves important functions. For example, the hairy tail in a kangaroo or dog facilitates its locomotion while the hairless tail in beaver helps regulate its body temperature^1, 2^. Despite such roles of tail, tail-loss can be observed in many animals^3, 4^. To gain insight for the tail-loss, several studies investigated genes responsible for this change. One of the studies reported that a non-synonymous point mutation, in conserved T-box domain of the T gene, causes shortening of tail^5^. Additionally, fundamental researches on the posterior development may also provide a clue for the tail-loss. For example, a study on developmental biology describes TBX family, which formed as a result of duplication of T gene^6^. TBX6 of the TBX family was found to participate in differentiation of posterior stem cells during embryo development^7^. The absence of TBX6 was reported to induce neural plate and somite development^8, 9^. This implies that TBX6 regulates differentiation of posterior stem cells which form a tail in the later stages. Apart from T gene and TBX family, various other studies attempted to discover other causal gene for the short tail including HES7^10^.

Although many studies investigated tail-loss, the majority of the researches were done in commonly used model animals such as mouse. It is impossible to explain the loss of tail in other species with the mouse model alone due to variation between species; hence more studies on other non-model organism are required to provide better understanding of the tail-loss. One of the preceding works on non-model organisms investigated shortening of tail in Pembroke Welsh Corgi^11^ which is a representative dog breed with short tail, while another study analysed tail-loss in 23 different dog breeds^4^. These studies suggested that a non-synonymous variant in T gene causes the short tail, however, it did not apply to all the dog breeds with short tail.

The aim of this study is to investigate tail-loss in a traditional Korean dog, DongGyeongi (DG). DG is an endangered breed with short tail which is being protected as a natural monument in Korea since 2012 (Cultural Heritage Administration of Korea, number: 540). Various reports of this particular breed including old Korean records, Dongkyung jabki (published in AD 1669) and Sungho sasul (published in AD 1740) as well as the clay dolls of DG excavated from the remains of the ancient Kingdom, Silla proves that this breed has been bred in Korea for at least 1,000 years.

In this study, 22 blood samples of DG were sequenced and genotyped. Using this data, population structure and phylogenetic tree analyses with other dog breeds were performed. Finally, variant analysis was carried out to discover putative causal variants for tail-loss. The finding in this study suggests possible evolutionary history of short tail phenotype and it also provides candidate variants that may lead to short tail in DG as well as other dog breeds.

## Results

### General Features of DongGyeongi

**Figure 1:**
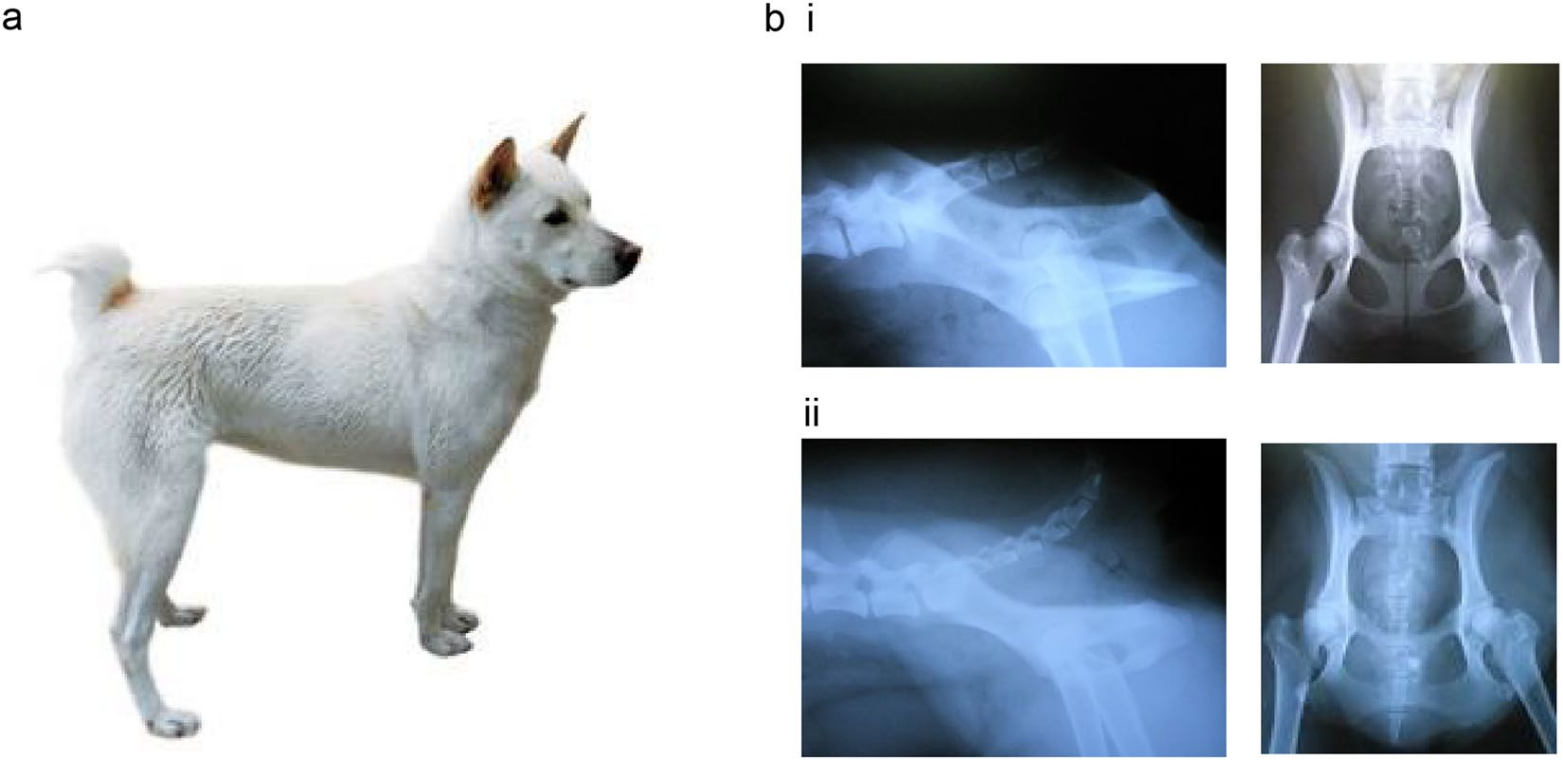
DongGyeongi (DG) and X-ray of DG’s tail. (**a**) shows the general appearance of DG. (**b** i) The tail length of 2 coccygeal bones can be observed from the Fig. (**b** ii) is the tail of a short tail sample. The tail length of 5 coccygeal bones can be observed in the figure. The samples shown in this figure are not used in this study, but they represent the common characteristic tail length for no tail and short tail groups. *Gyeongju dog DongGyeongi BaekGu (the name of the owner: Korean Gyeongju DongGyeong Dog Association Co.)’, by ‘ Korean Gyeongju DongGyeong Dog Association Co.’ in 2012 under Type 1 license of the KOGL. This item can be downloaded free in ‘Cultural Heritage Administration.

Figure 1a shows the appearance of DG. In general, the weight of adult male and female DG are 16–18 kg and 14–16 kg respectively. The withers height and body length of a male are typically 47–49 cm and 52–55 cm while those of a female are 44–47 and 49–52 cm. DG can be categorized into three groups depending on their tail length. One of the three groups is no tail group (NT) which typically has 2~3 coccygeal bones. On the other hand, short (ST) and long tail groups (LT) possess 5~7 and ~20 coccygeal bones, respectively. Figure 1b which shows X-ray of tails of DG demonstrates such general features of DG groups. Figure 1b i which represents NT shows the tail length of 2 coccygeal bones, while the Fig. 1b ii representing ST displays the tail length of 5 coccygeal bones, distinguishing the two groups.

### Re-sequencing and Population Structure Analysis of Dong-Gyeongi

**Figure 2:**
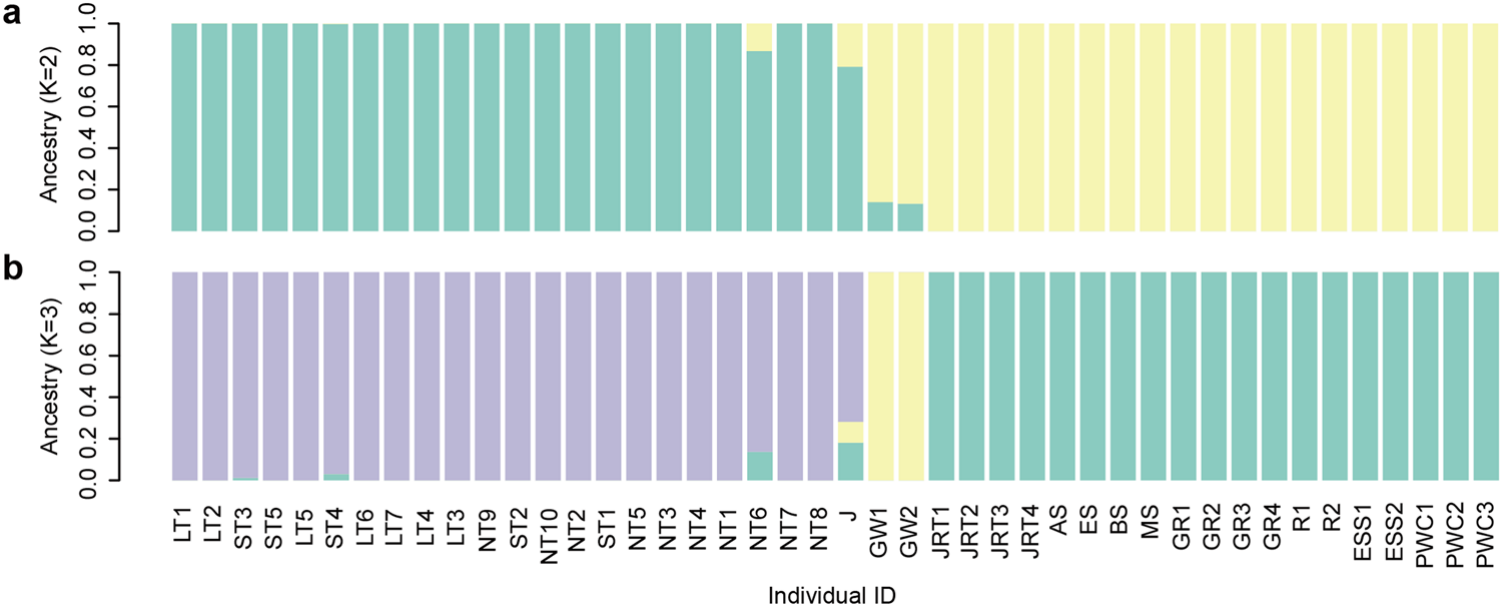
The population structure of DongGyeongi (DG) samples and other public dog data. (**a**) The bar plot on the top represents the structure when the number of assumed ancestral population of K = 2, while (**b**) the bar plot below it shows the structure at K = 3. The different colour of the bar chart denotes the structure of a different ancestral population. *LT: long-tailed DG, ST: Short-tailed DG, NT: no-tailed DG, J: Jindo, GW: Gray wolf, JRT: Jack Russell Terrier, AS: Australian Shepherd, ES: English Setter, BS: Brittany Spaniel, MS: Miniature Schnauzer, GR: Golden Retriever, R: Rottweiler, ESS: English Springer Spaniel, PWC: Pembroke Welsh Corgi.

The summary of the re-sequencing data is presented in the supplementary Table S1. Average alignment rate of 98.87% and the depth of over 15X were achieved from the sequencing data of DG. As a result of variant calling step, 5,682,193 SNPs, 1,365,208 insertions and 1,281,640 deletions were identified. Using the randomly selected subset (~130,000) of the identified variants, the population structure analysis was conducted on 11 dog breeds and gray wolf to investigate if DG breed has a unique genetic structure. Figure 2a shows the admixture of each of the dog breeds when the analysis was conducted with the number of assumed ancestral population (K) of 2 which showed the least cross validation error (Supplementary Fig. S3). The structure of the 22 DG and a Jindo samples shows similar pattern, while that compared to other dog breeds was clearly distinguishable. Figure 2b shows the structure of dog breeds when the K value is 3. Again the genetic structure of DG and Jindo shared some similarity while they were completely different when compared to other dog breeds. In addition, the genetic structure of gray wolf which is one of the closest species to dog^12^, diverged from the rest of dogs earlier than any other dog breeds, showed distinct structure compared to the structure of the other dog breeds.

### Evolutionary History of the tail-related Gene of DongGyeongi

**Figure 3:**
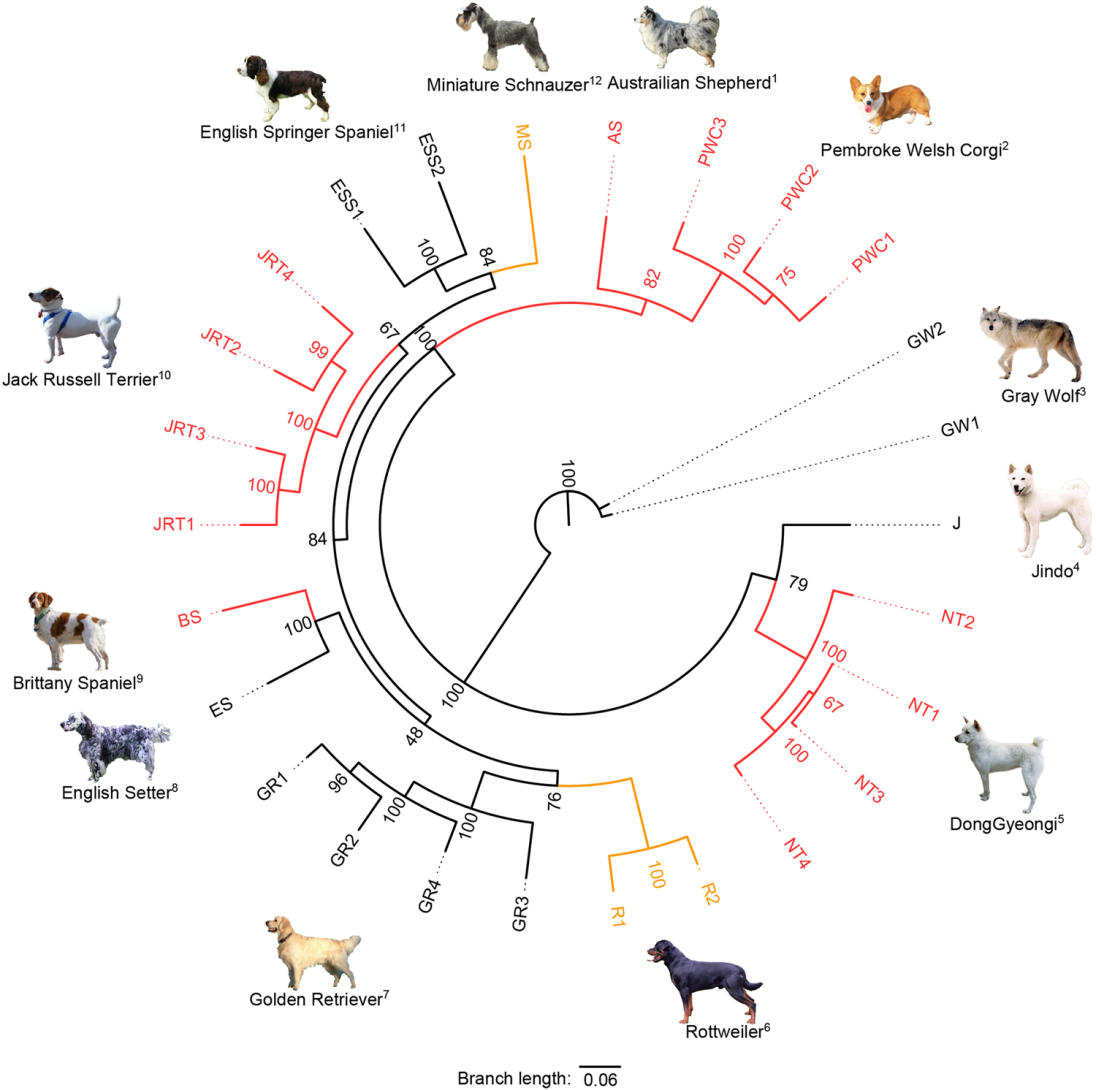
The phylogenetic tree based on the whole genomic SNPs of different dog breeds. The colour red represents the breeds with short-tail phenotype, while the colour yellow denotes for the short-tailed breeds with unknown causal variants. The breeds coloured in black are the normal dog breeds with less frequent occurrences of the short tail phenotype. The number written in each node represents the bootstrap value. ^1^Figure obtained from https://commons.wikimedia.org/wiki/File:Australian_Shepherd_Blue_Merle.jpg. ^2^Figure obtained from https://en.wikipedia.org/wiki/File:Welsh_Corgi_(3452560074).jpg. ^3^Figure obtained from https://www.flickr.com/photos/usfwsmidwest/6545954933/in/set-72157628504266513/. ^4^Figure obtained from https://commons.wikimedia.org/wiki/File:Korean_Jindo_Dog.jpg. ^5^‘Gyeongju dog DongGyeongi BaekGu (the name of the owner: Korean Gyeongju DongGyeong Dog Association Co.)’, by ‘ Korean Gyeongju DongGyeong Dog Association Co.’ in 2012 under Type 1 license of the KOGL. This item can be downloaded free in ‘Cultural Heritage Administration’. ^6^Figure obtained from https://commons.wikimedia.org/wiki/File:Rottweiler_300rt.jpg. ^7^Figure obtained from https://commons.wikimedia.org/wiki/File:Golden_Retriever_Dukedestiny01_drvd.jpg. ^8^Figure obtained from http://thepetwiki.com/wiki/File:English_Setter.jpg#filelinks. ^9^Figure obtained from https://commons.wikimedia.org/wiki/File:Brittany_Spaniel_standing.jpg. ^10^Figure obtained from https://commons.wikimedia.org/wiki/File:Jack_Russell_Terrier_in_Park.jpg. ^11^Figure obtained from https://commons.wikimedia.org/wiki/File:EnglishSpringerSpan2_wb.jpg. ^12^Figure obtained from https://commons.wikimedia.org/wiki/File:Miniature_Schnauzer_02.jpg. ^1,4,6,8–12^are under Creative Commons Attribution-Share Alike 3.0 Unported license (https://creativecommons.org/licenses/by-sa/3.0/us/). ^2,3^are under Creative Commons Attribution-Share Alike Attribution 4.0 International license (https://creativecommons.org/licenses/by-sa/4.0/).

The phylogenetic tree was constructed to investigate the ancestral history of the DG and 10 other dog breeds that were categorized into 3 groups according to their length of tail and probable cause of that phenotype (Fig. 3). These groups were represented by different colours. The taxa coloured in red including Australian Shepherd, Pembroke Welsh Corgi, Brittany Spaniel and Jack Russell Terrier are the ones that frequently show the variant in the coding region of T gene along with the short tail phenotype^4^. The breeds expressed by yellow including Miniature Schnauzer and Rottweiler indicates dogs with the short tail phenotype caused by the unknown genetic factors according to a previous study^4^. Finally the remaining dogs in black represent the breeds which show less frequent short tail. Moving onto the position of each taxon, the individual samples from each dog breed formed a clustered taxon representing that breed.

The general structure of the tree is similar to the tree previously generated by other studies^13^, although the position of some breeds within the tree does not completely match. For example, Pembroke Welsh Corgi was clustered with Australian Shepherd (Fig. 3). Also, these two breeds located themselves outside the cluster of Golden Retriever, Rottweiler and Jack Russell Terrier similar to the tree topology in other studies^13^; however, including the position of the two Spaniel breeds which does not show the closest evolutionary distance, several differences of the tree compared to the previous tree can be observed. In addition, DG and Jindo which are Korean dog breeds showed the significant distance from the rest of the dog breeds similar to the observation from their population structure in Fig. 2. The short tail breeds that frequently show T gene variants, including DG, Austrailian Shepherd, Pembroke Welsh Corgi, Brittany Spaniel and Jack Russell Terrier did not form a monophyletic branch. Also, short-tailed breeds with unknown genetic cause including Miniature Schnauzer and Rottweiler did not form a monophyletic branch.

### Demographic analysis of DongGyeongi population

Demographic analysis was conducted using (G-PhoCS) to estimate divergence time of DG population with migration and with the absence of migration. Assuming the average mutation rate as 1 × 10^–8^ and average generation time of 3 years, divergence time estimate of DG from indigenous Korean dog, Jindo with and without gene flow were 923 (CI of 0–1,933) and 965 (CI of 0–2,114) years respectively (Table 1). Moreover, ancestor of Jindo and DG was found to be diverged from wolf approximately 25,000 years ago. Effective population size of the Ancient wolf was found to be ~50,000 which decreased to ~6,000 in Gray wolf and ~13,000 in ancient Korean dog. The effective population size of Korean dogs decreased further to ~5,000 in DG and ~2,000 in Jindo. Finally moving on to the rate of gene flow, the highest rate was observed by the gene flow of wolf to ancient Korean dog, followed by the flow from ancient Korean dog to wolf.

**Table 1:** Demographic parameters estimated by G-PhoCS.

	With migration	Without migration
θDG	5,776 (0–11,732)	5,909 (0–12,255)
θJ	1,869 (0–3,870)	1,946 (0–4,243)
θGW	6,445 (5,718–7,171)	6,464 (5,718–7,172)
ΘAKD	13,695 (12,081–15,310)	13,720 (12,081–15,352)
ΘAW	50,546 (49,362–51,729)	50,527 (49,362–51,689)
TDG	923 (0–1,933)	965 (0–2,114)
TAKD	25,491 (22,561–28,420)	25,563 (22,561–28,406)
mDG > J	0.0010 (0–0.0056)	n/a
mJ > DG	0.0014 (0–0.0088)	n/a
mAW > AKD	0.4134 (0–3.3092)	n/a
mAKD > AW	0.0233 (0–0.2000)	n/a

*θ: effective population size, T:divergence time, m: gene flow rate, DG: DongGyeongi, J: Jindo (indigenous Korean dog), GW: Gray wolf, AKD: ancient Korean dog, AW: ancient wolf. The values in the parenthesis are 95% confidence interval of the estimated parameters.

### Selective sweep region of DongGyeongi population

**Figure 4:**
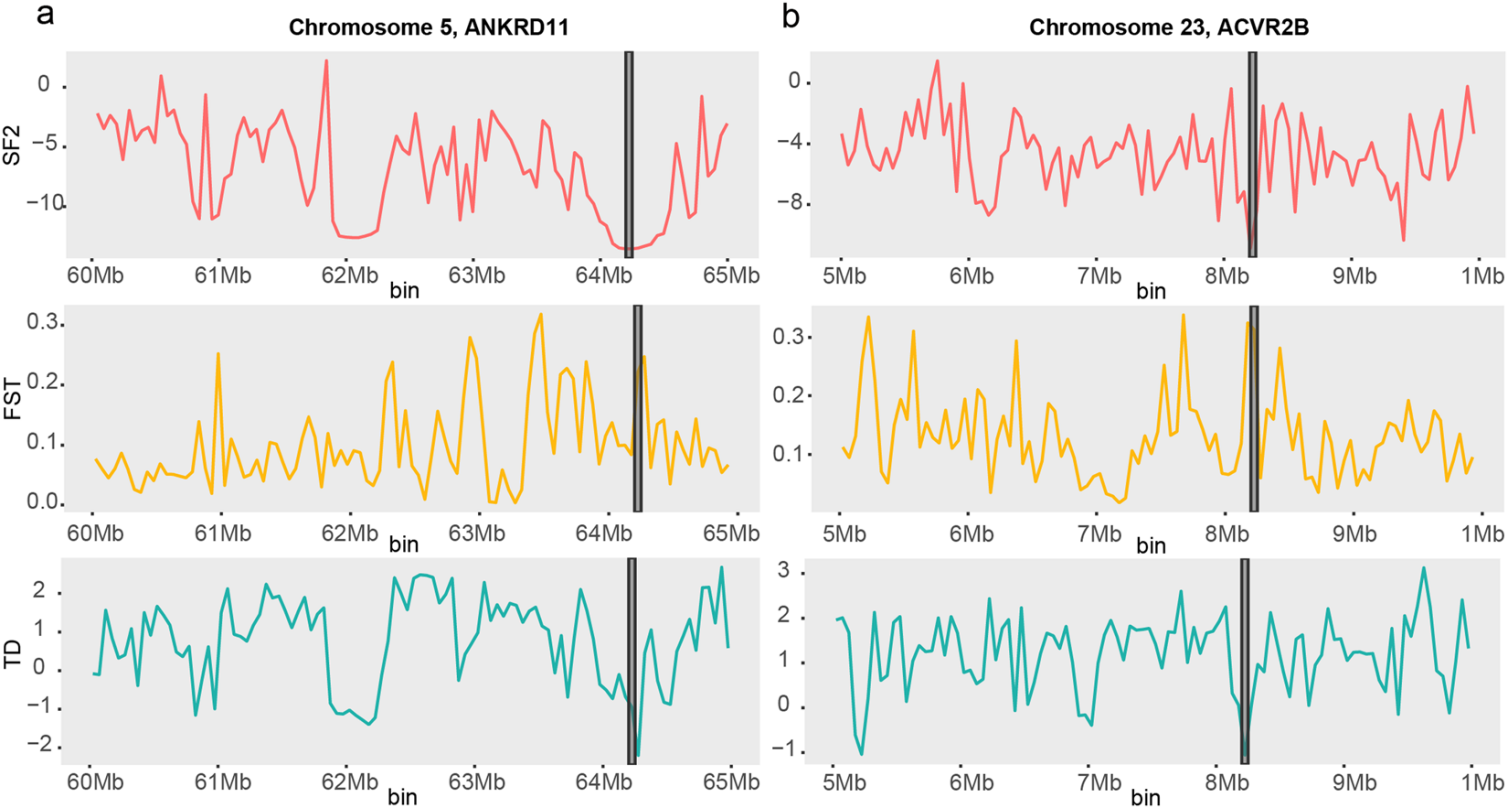
Identification of candidate selective sweep genes. The candidate genes identified by selective sweep analysis including SweepFinder2 (SF2), Fst and Tajima’s D located in in the highlighted box in grey are shown. Significantly low SF2, Tajima’s D and high Fst values indicating selective sweep signature were detected.

As a result of selective sweep analysis using combination of evidences including SweepFinder2, Fst and Tajima’s D, 120 of 50k bins were identified which contained 72 genes in them (Table S3). Among the identified genes, ANKRD11 and ACVR2B are known to be associated with skeletal system development (Fig. 4). ANKRD11 was discovered in the candidate selective sweep region of 64.2–64.25 Mb bin in chromosome 5. It was reported that mutation or microdeletion in this gene cause KBG syndrome which gives rise to skeletal malformation in human^14, 15^. At the same time, ACVR2B found in 8.2–8.25 Mb bin of chromosome 23 was known to be associated with differentiation of bone marrow stromal cells^16^.

### Candidate Variants responsible for Tail-loss and Nucleotide Diversity near the Variants

**Figure 5:**
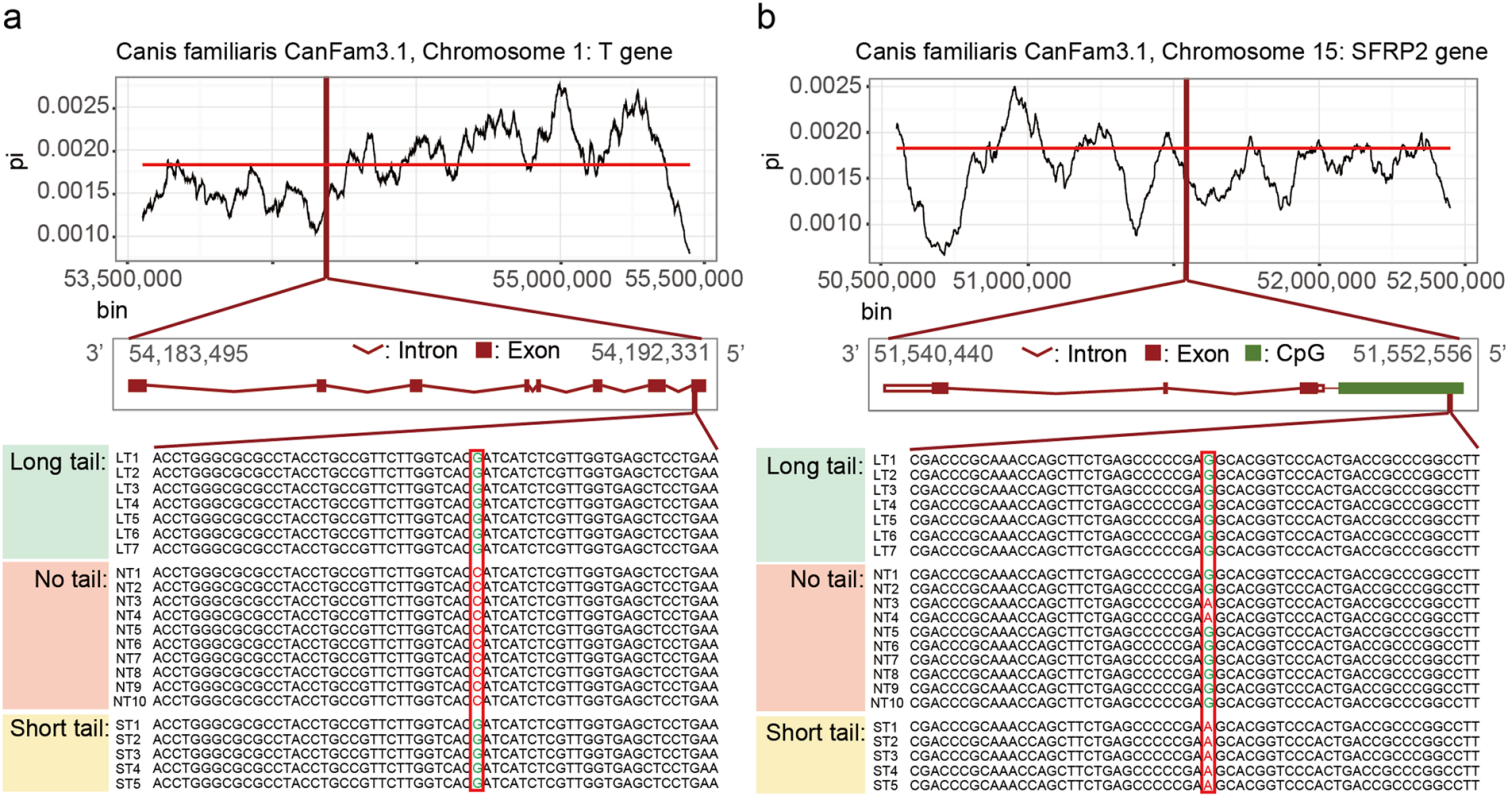
The candidate variants identified as a result of the variant analysis. (**a**) The T gene variant located in the coding region of the T gene. The line graph on the top shows the nucleotide diversity profile across 2MB region near the gene. The aligned sequence below displays the variant which specifically occur in no-tailed DongGyeongi (DG) samples. (**b**) The CpG island variant located near the SFRP2 gene. Below the nucleotide diversity profile of 2 MB regions near the gene, it shows the mutually exclusive variants between short-tailed and long-tailed DG samples.

To identify the causal variant for tail-loss in DG, variant analysis was performed. Among 4,717 single nucleotide variants (SNVs) whose genotypes were found mutually exclusive in the NT as compared to the LT. 15 SNVs were located in the coding sequence. Furthermore, 3 out of the 15 SNVs, including T, ZNF329 and Aldh7a1 gene variants were non-synonymous variants (Table S4). Out of these 3 variants, the one located in T gene was heterogeneous while the rest of the variants were mixed by heterogeneous and homogeneous genotypes. On the other hand, none of ST-specific variants was observed in the coding region. When regulatory regions were taken into consideration, 4 SNV and 3 indel variants located in CpG islands, as well as one SNV in intron were identified to be ST-specific (Tables S5 and S6). All SNVs were heterogeneous while 2 of 3 indels were heterogeneous variants. Through the thorough literature research on these variants, this study suggests a SNV in the coding region of T gene and another SNV in the CpG island of SFRP2 gene to be the candidate causal variants respectively for NT and ST phenotypes (Fig. 5).

To investigate sequence conservation near the candidate variants for tail-loss, the nucleotide diversity of the sequence was calculated near the variants. Amongst the identified variants, Fig. 5 shows nucleotide diversity near the T and SFRP2 gene. The nucleotide diversity near the T gene as shown by the highlighted rectangle, is slightly reduced, in comparison to the average nucleotide diversity of 0.00183 of the 2MB bin (Fig. 5a). Towards 3’ direction of the variant, the nucleotide diversity dropped, whereas this value reaches a peak in the 5’ end direction. The nucleotide diversity near the SFRP2 gene was also found to be slightly reduced (Fig. 5b), compared to the average nucleotide diversity of 0.00160 across the 2MB region.

## Discussion

DG is the oldest and endangered dog breed in Korea (Dongkyung jabki, AD 1669, Sungho sasul, AD 1740) that the population of DG and its genetic information is maintained and preserved by the Korean government (Cultural Heritage Administration of Korea, number: 540); about 600 DG are currently being protected in Korea. Amongst the current DG population, the number reduces further to approximately 200 for NT DG. Therefore, the sequencing of 22 DG used in this study not only represents significant proportion of the current genetic pool of the entire DG population (Supplementary Figs S1 and S2) but it also will prevent complete loss of DG’s genetic information. Moreover, DG is also the only dog breed in Korea that has short tail, and unlike other non-Korean breeds with short tail, its tail can be classified into several groups due to the variable length. Therefore, DG is a remarkable breed for the researches on tail-loss. In this study, DG was investigated in order to predict the evolution of short tail phenotype and to identify the variant responsible for tail-loss. Whole genome sequencing was performed for 22 samples of DG followed by investigation of population structure, phylogenetic tree, demographic history, selective sweep and causal variant for the short tail phenotype.

For the population structure analysis, the distinct genetic structure of DG was observed compared to other dog breeds. The population structure analysis was conducted for each of the K values ranging from 2 to 12. Figure 2 shows the population structure of different dog breeds when K is 2 and 3 since the least error is produced by these K values. As a result of the population structure analysis, it was concluded that the general pattern of DG was clearly different from the other dog breeds except for Jindo which shares the regional origin with DG. This observation shows the distinguishing breeding line of the DG and other Korean breed which was isolated from other dog breeds for a long period of time. In addition, the population structure analysis at higher values of K showed high variation of genetic structure between DG samples (Fig. S4–1 and S4–2). This was an unexpected observation because inbreeding is known to be practiced between short-tailed DG. From this result, it can be inferred that extensive recent gene flow events into the DG population may have taken place before inbreeding was initiated. Such an event can also be the reason for relatively higher effective population size of DG compared to that of Jindo (Table 1).

Phylogenetic tree analysis was done to provide the general ancestral history of short tail in modern dog breeds (Fig. 3). Since the resulting tree was constructed using a set of the strictly filtered SNPs from the whole genome, its structure was not identical to the tree constructed by another study, which used different subset (~48,000) of SNPs^13^; however, note that some parts of the two trees also shared similar topology. According to the tree constructed in this study, DG and Jindo are predicted to have diverged from the rest of the dog breeds earlier than any other breeds (Fig. 3), converging into the same conclusion drawn by the population structure analysis. To be more specific, the demographic analysis conducted showed that the DG was diverged from Jindo approximately 1,000 years ago (Table 1). Regardless of such ancestral history, the short tail phenotypes are observed in DG and other dog breeds including Austrailian Shepherd, Pembroke Welsh Corgi, Brittany Spaniel and Jack Russell Terrier which produce short-tailed offspring with T gene variant. These dog breeds, do not form a monophyletic group and it is unlikely that these dog breeds share the most recent common ancestor which passed onto them the short tail phenotype. Likewise, the previously suggested dog breeds including Miniature Schnauzer and Rottweiler which produces short tail without the T gene variant do not form a monophyletic group either. These breeds are predicted to have developed short tail independently.

Based on these findings, we can suggest two possible explanations for the evolution of short tail. First, reduction of tail length in DG may have been developed as a result of gene flow from other dogs with short tail. Because DG is the only dog in Korea with short tail, it is possible that there had been interbreeding event with another tail-less dog from abroad before the first record of DG during the ancient era of three kingdoms of Korea. One of the three kingdoms, Silla in which DG originated, had been actively trading with other countries outside the East Asia^17^. Hence, there may have been gene migration from tail-less dogs brought by the foreign traders into the ancestor of DG. Note also that the divergence time of approximately 1,000 years of DG found in the demographic analysis does not violates this theory (Table 1). Another possibility of DG developing short tail phenotype is by the result of convergent evolution. Dogs born short-tailed may have been preferred by some owners. This may have led to selection for those DG with short tail. In this way, the convergent evolution by artificial selection may have resulted in formation of dog breeds with short tail throughout the world. The reduction in the effective population size of ancient Korean dog breeds to DG may be an evidence of such selection event.

With the possible evolution of tail-loss in DG suggested, we looked into more details on the genotypes that are associated with the short tail. To find the genetic factors underlying DG’s short tail, selective sweep analysis and variant analysis were conducted based on the genetic information obtained from the re-sequencing of DG genome. As a result of selective sweep analysis, genes including ANKRD11 and ACVR2B which was reported to take part in skeletal system development were identified (Fig. 4). Although there has not been an experimental validation conducted on dogs, the strong selection signature observed near the two genes which are important components of bone stromal cell differentiation and other pathways associated with bone malformation, indicates that they may contribute to the tail-loss.

Moving onto the variant analysis, we hypothesized that tail-loss is caused by one dominant allele. This is because it has been known that long-tailed DG can be born from the two parents with short tail phenotypes, and DG population contains small proportion of long-tailed individuals even though inbreeding has been practiced amongst short-tailed DG for a long time. As a result of the analysis on NT, a non-synonymous and heterogeneous point variation in the coding region of T gene was identified. T gene is known to code for T transcription factor which contains T-box conserved domain. This domain is known to be conserved in many species ranging from invertebrates to vertebrates^18, 19^. Moreover, it has been also reported that T transcription factor is associated with the posterior mesoderm development in many animals including evolutionarily close species, mouse^20, 21^. Since a variant discovered in DG samples is located within the conserved T-box domain of T gene, it is likely that this variant also involves in posterior mesoderm development or other developmental process crucial for tail development in dog, leading to loss of tail in NT DG samples. More importantly, the T gene variant identified in this study is identical to the variant associated with the short tail suggested by the previous studies on other dog breeds^4, 11^. Similar to these studies, the variant found in DG is predicted to interfere with the tail formation during the developmental stage of dog embryo, through reducing the affinity of T transcription factor towards its downstream target DNA sequence^11^. Combined with the experimental validation on T gene variant provided by the previous study^3^, this study suggests the same variant to be the putative causal variant for the tail-loss in DG.

For the analysis of ST, similar approach could not identify any variant located in the coding sequence, which indicates that the decrease in the tail length of ST samples is not due to the change of a gene’s product. Therefore, another possibility we looked into was the gene regulation. The regulatory regions and CpG islands that may be responsible for gene expression level were analysed. As a result, a variant located in the CpG island of SFRP2 gene was identified. SFRP2 gene is activated at the developmental stage of embryo. Knockout of this gene is known to cause defects in cell migration, resulting in elongation of anteroposterior axis while decreasing in the posterior axis^4, 22^. Similarly, the CpG island variant of SFRP2 gene in the ST samples may result in decreased expression of this gene. This can lead to decreased cell migration rate during the developmental stage of embryo which in turn reduces DG’s tail length. However, the function of SFRP2 gene needs to be validated in dogs. Such a validation step can be done by demonstrating changed phenotype in dog embryo when the point mutation is present. Due to technical limitation and difficulty of obtaining more DG samples, it is practically impossible to conduct this kinds of validation in short period of time. Hence, further functional validation of this gene and variant will be one of the future directions of this study.

Based on the two variants suggested nucleotide diversity of the neighbouring regions was investigated to detect if there is selection signal. The nucleotide diversity near the two candidate variants were found to be slightly reduced compared to the average nucleotide diversity of the 2MB bin (Fig. 5). Such a reduction in variability can be observed if individuals showing certain phenotype associated with that region are selected. Therefore, the reduced nucleotide diversity near the variants may indicate artificial selection of DG with short tail even though the signature was not strong enough to be discovered in the selective sweep analysis. The functional importance of T and SFRP2 gene may also contribute to the reduced nucleotide diversity.

Overall, we suggest the two possible causes of DG’s short tail are: 1) from admixture with non-Korean dog that has short tail or 2) as a result of convergent evolution, supported by the population structure and phylogenetic tree analysis. Moreover, we also suggest ANKRD11 and ACVR2B genes located near selective sweep region, the variant in the coding region of T gene and the CpG island of SFRP2 to be the candidate genetic factor responsible for tail-loss in DG.

## Materials and Methods

### Sampling and Sequencing of 22 DongGyeongi DNA

The current study used 22 samples of DG. These samples were classified into three groups according to their tail length. Ten of the DG samples with short tail length (2~3 coccygeal bones) were defined as the ‘no tail’ group (NT), while five (5~7 coccygeal bones) and seven (~20 coccygeal bones) of the DG samples were cartegorised into ‘short tail’ (ST) and ‘long tail’ groups (LT), respectively.

DNA was extracted from the blood samples of DG. Indexed shotgun paired-end libraries of insert length ~500 bp were prepared for the DNA samples, using TruSeq Nano DNA Library Prep Kit (Illumina, USA). The standard Illumina sample-preparation protocol was followed in this step. The genomic DNA of ~200ng were then further fragmented into smaller piece of ~500 bp, using Covaris M220 (Woburn, MA, USA). End repair, A-tailing and adapter ligation (~125 bp adapter) was performed followed by gel-based size selection for 550~650 bp. The DNA was amplified by 8 cycles of PCR. Agilent 2100 Bioanalyzer (Agilent Technologies) was used to analyse the size distribution of the DNA and to detect contamination. Finally, the resulting DNA were sequenced by Illumina HiSeq. 2500 sequencer (2 × 125 bp paired-end reads were generated).

All methods were performed in accordance with the guidelines and regulations provided by the Committee on Ethics of Animal Experiments (CEAE), National Institute of Animal Science, Republic of Korea (Permit Number: NIAS2015–774). The DNA extraction protocol was approved by the CEAE. Genomic DNAs were extracted from blood samples obtained from the Institute of Conservation Gyeongju DongGyeongi in Republic of Korea with permission.

### Re-sequencing and Variant Calling of DongGyeongi Genomes

The quality of each base of the paired end reads was visualized using the fastQC (http://www.bioinformatics.bbsrc.ac.uk/projects/fastqc/). The adapters and unpaired read sequences from the whole genome sequencing step were removed by Trimmomatic-0.33^23^ before mapping the reads against the dog reference genome CanFam3.1, using Bowtie2–2.2.5^24^. For downstream processing of the mapped reads, the reads were first grouped using Picard 1.138 (http://picard.sourceforge.net), based on different lane in which they were generated, to compensate for the variable environment. Also, the duplicated reads from PCR were filtered using Picard. Following the removal of duplicates, local realignment was performed to correct errors caused by insertions or deletions using Genome-AnalysisTK-3.4–46 (GATK)^25^. GATK was also used for SNP and indel calling. Criteria used for the variant calling include (1) count of reads that have mapped quality 0 (MQ0) > = 4 and (MQ0/DP) > 0.1, (2) variant call confidence (QUAL) < 30, (3) QUAL score normalized by allele depth (QD) < 5.0 and (4) strand bias score from Fisher’s Exact test (FS) > 200.

### Population Structure Analysis of Dog Breeds

Population structure analysis was conducted using ADMIXTURE^26^ with 10 more dog breeds including those whose tail shorten with T gene variant, similar to NT of DG (Australian Shepherd, Brittany Spaniel, Jack Russell Terrier and Pembroke Welsh Corgi), short tail breeds without T gene variant (Miniature Schnauzer and Rottweiler), and long tail breeds with less frequent T gene variant occurrence (English Setter, English Springer spaniel, Golden Retriever and Jindo dog) which were downloaded from NCBI SRA database (Table S2). Based on the whole genomic SNP variants of the 44 samples, approximately 130,000 SNPs were randomly selected using PLINK ‘–thin’ option^27^. The population structure analysis was conducted with each of K values ranging from 2 to 11 using the bootstrap of 1,000.

### Phylogenetic Tree Analysis of Dog Breeds

Phylogenetic tree analysis was conducted by SNPhylo software package^28^. The 11 dog breeds including DG used in the population structure analysis were used for this analysis. However, when all DG samples were used for the tree construction, the resulting tree was not possible to represent ancestral tree of domestic dogs due to the difference in sample size between DG samples and other dog breeds, which introduced bias during the SNP filtering step. Therefore only 4 samples of DG were used to construct the phylogenetic tree of different dog breeds. Moreover, two gray wolf samples were used as an out group to estimate root of the tree. Maximum likelihood tree was constructed based on the filtered 15,064 SNPs representing each LD block (LD > 0.1, MAF > 0.1) and, using the bootstrap value of 1,000. FigTree program was used to visualize the tree.

### Demographic inference using G-PhoCS

Generalized Phylogenetic Coalescent Sampler (G-PhoCS) was used for demographic history analysis of DG population^29^. G-PhoCS estimates various demographic parameters based on neutrally evolving and independent loci using the probabilistic model of coalescent with migration and Markov Chain Monte Carlo (MCMC) sampling strategy. First of all, the whole genome sequences of DG, Korean indigenous dog, Jindo and Gray wolf were filtered to obtain neutral loci. Gaps and regions with low map quality score (Q < 10 of samtools) in CamFam3.1 genome were filtered out. Moreover, repeats detected by repeatmasker (http://repeatmasker.org) (score > 25), protein coding sequences (annotated by Refseq and Ensembl) and their flanking 10 kb which may have been influenced by natural selection were filtered out as well as conserved non-coding elements in euarchontoglire mammals shown by the 30-way alignment and the flanking 100 b regions. Regulatory regions defined by Oregano database^30^ and CpG island downloaded from UCSC were further removed. To obtain independent fragments of DNA, we selected 1 kb of loci every 50 kb. Finally, 19,113 of such loci were identified.

For G-PhoCS analysis, we used a subset of 6,371 loci by selecting one in every three of the neutrally evolving loci to expedite the process. The default parameters used in the analysis were as following: Gamma distribution with α = 1 and β = 10,000 for population size and divergence time scaled by mutation rate, and gamma distribution with α = 0.002 and β = 0.00001 for the migration rates scaled by mutation rate. The Markov Chain was run for 1,200,000 iterations, 500,000 of which were treated as burn-in. Every 100 iterations, parameter values were collected and as a result total of 70,000 samples were obtained to estimate those parameter values.

### Selective sweep analysis of DG samples

Selective sweep analysis was conducted using sweepfinder2 to detect candidate selective sweep region based on composite likelihood ratio (CLR) statistics^31^. The ancestral genotypes of DG were estimated using Gray wolf as outgroup. Subsequently, sweepfinder2 –f option was used to calculate background site frequency spectra of all autosomal chromosomes, before carrying out scan for selective sweeps.

Moreover, Fst and Tajima’s D statistics were also calculated to provide evidence of recent selection using VCFtools (v0.1.13). The VCFtools implementation of Fst measures the degree of population differentiation based on allele frequency of different populations^32^. The resulting Fst value of 0 can be observed between populations which can interbreed freely while the value of 1 is observed between populations which do not share any genetic diversity. Hence we considered high Fst value as an evidence of selection process since selective sweep give rise to highly differentiated regions. Tajima’s D statistics measures the difference between observed genetic variation and the expected variation to investigate balancing selection and positive selection^33^. The negative value of Tajima’s D signifies positive selection while the positive value indicates balancing selection. In this study, Fst and Tajima’s D statistics of window size of 50k were calculated to support selective sweep region identified by sweepfinder2. The regions showing the top 5% statistics of all tests were considered strong candidates of selective sweep.

### Identification of the putative causal Variants related to the NT and ST

The different causal variants for NT and ST phenotypes were hypothesized, and hence, identification of these variants was conducted separately for the two sample groups (NT and ST). For NT variants, mutually exclusive variants compared to the LT were selected during the variant analysis and amongst these variants, the variants located in the coding regions were considered. Synonymous variants which are not likely to cause change in the resulting protein structure, were further filtered out to select candidate causal variants. In case of ST causal variant, regulatory regions defined by ORegAnno^30^, and CpG islands^34^, which may alter expression level of genes were also considered.

### Nucleotide Diversity near the Candidate Variants

Nucleotide Diversity of all samples was calculated by vcftools (v0.1.13), across the 10 Mb region near the two candidate variants^35^, to determine the heterozygosity near the candidate variants in T and SFRP2 genes. For this calculation step, 100 kb of windows and 500 b of window step were used to visualize nucleotide diversity values across these regions.

### Availability of Data in NCBI Database

The DNA read data from DG samples were submitted to the NCBI database under BioProject ID of PRJNA358192.

## Electronic supplementary material

Supplementary information
