# Supplementary material for: The Genetic Origin of Short Tail in Endangered Korean Dog, DongGyeongi
Source: Sci Rep. 2017 Aug 30;7:10048. doi: 10.1038/s41598-017-10106-6 (PMC5577146; doi:10.1038/s41598-017-10106-6)
Supplement: Supplementary file 1 — Supplementary information [file 41598_2017_10106_MOESM1_ESM.pdf]

The genetic Origin of Short Tail in endangered Korean Dog, DongGyeongi

DongAhn Yoo[1,2], Kwondo Kim[1,2], Hyaekang Kim[3], Seoae Cho[1], Jin Nam Kim[1], Dajeong Lim[5], Seog-Gyu Choi[4], Bong-Hwan Choi[5]*, Heebal Kim[1,2,3]*

[1]C&K genomics, Seoul National University Research Park, Seoul, Republic of Korea

[2]Interdisciplinary Program in Bioinformatics, Seoul National University, Seoul, Republic of Korea

[3]Department of Agricultural Biotechnology and Research Institute of Agriculture and Life Sciences, Seoul National University, Seoul, Republic of Korea

[4]Institute of Conservation Gyeongju Donggyeong Dog, Dongguk University, Gyeongju 780-714, Republic of Korea

[5]National Institute of Animal Science, RDA, Wanju 565-851, Republic of Korea

*Corresponding author E-mail: Heebal Kim, (heebal@snu.ac.kr) and Bong-Hwan Choi (bhchoi@korea.kr)

Supporting Information

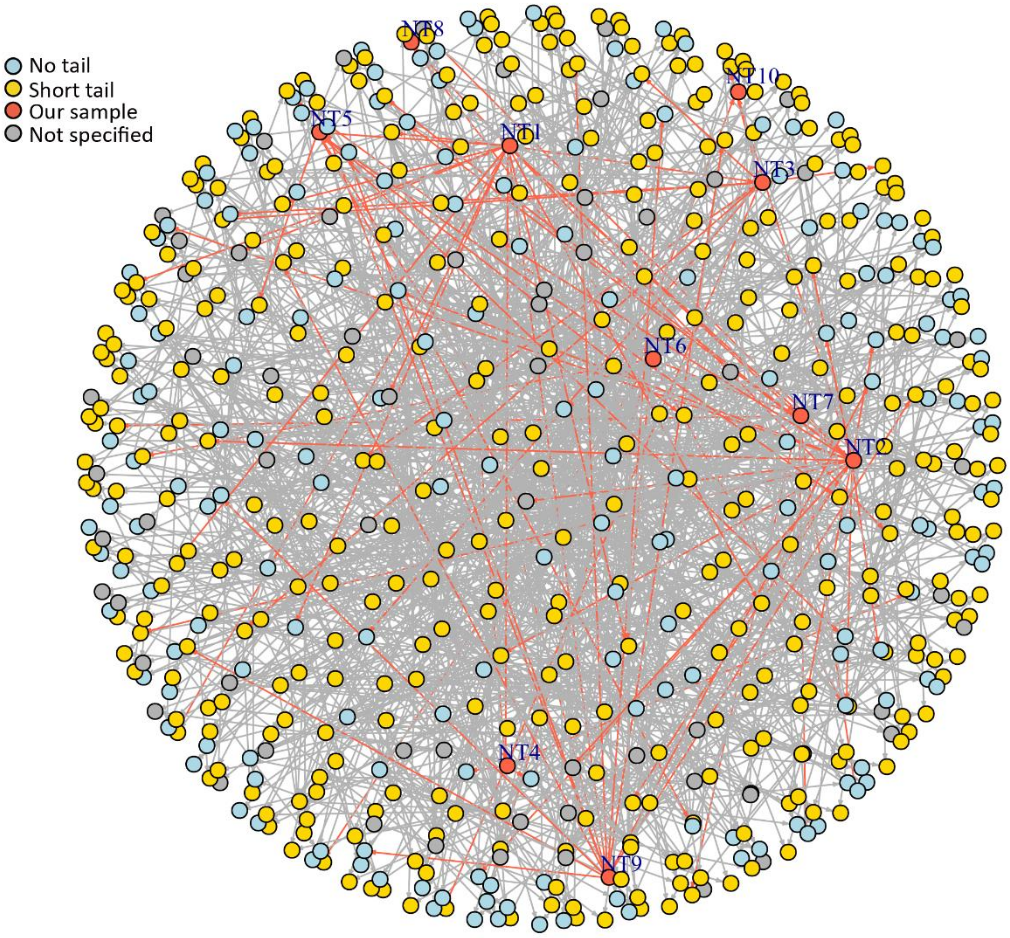

Supplementary S1 Fig. Network of DongGyeongi (DG) population currently living in Korea.

The blue and yellow nodes represent DG with no tail (NT) and short tail (ST), respectively. The red nodes indicates the NT samples used in the current study. The DG with missing tail-length information are denoted by the grey nodes. Each arrow shown in this figure points from the parent to offspring DG.

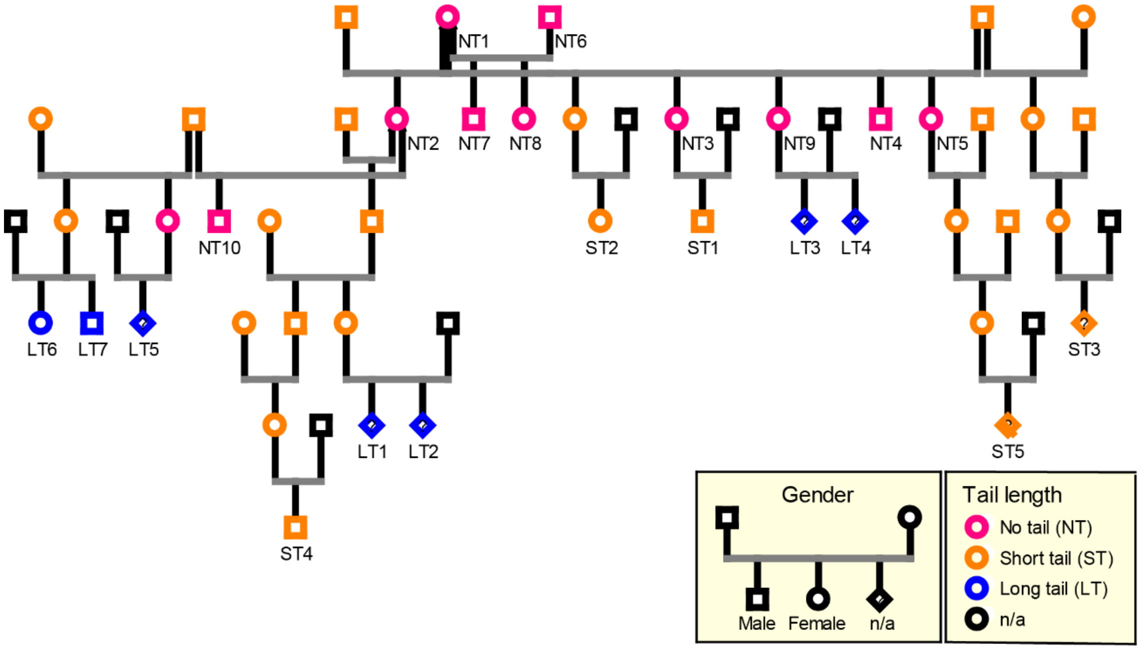

Supplementary S2 Fig. Pedigree of DongGyeongi (DG) samples used in this study. Rectangles and circles represent male and female, respectively. The samples with gender information missing are indicated by diamond symbols. The blue colour shows long tail (LT) sample used in this study, while the yellow and red denote short tail (ST) and no tail (NT) samples.

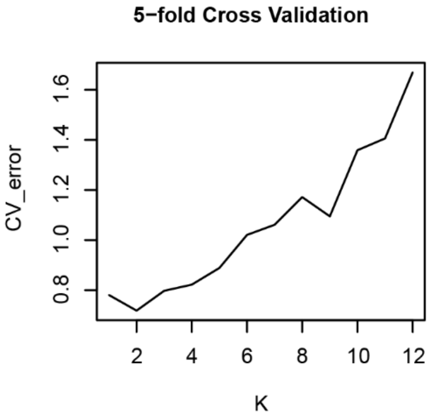

Supplementary S3 Fig. 5-fold cross validation of population structure analysis on 44 DongGyeongi and public dog samples.

The x-axis denotes the K value used in the population structure analysis while the Y-axis represents the cross validation error. Note that the cross validation error was the least at K = 2.

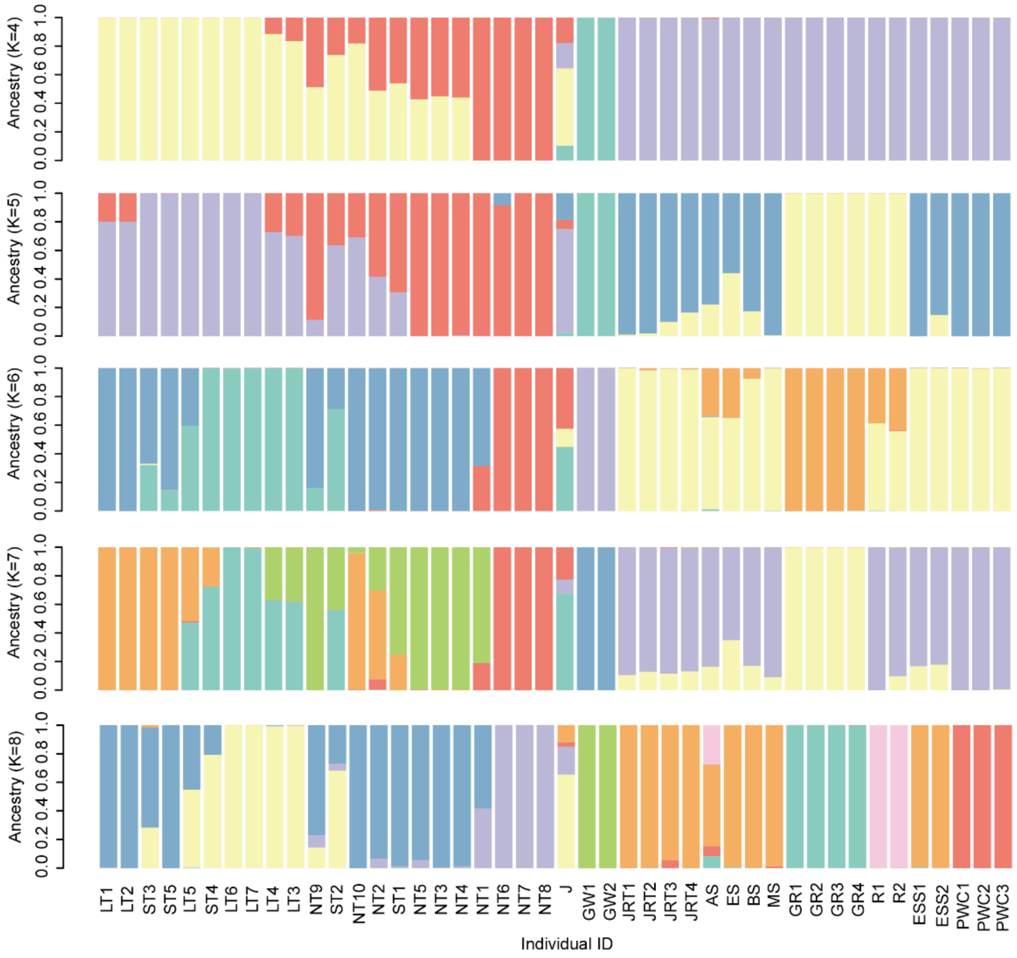

Supplementary S4-1 Fig. The population structure of DongGyeongi (DG) samples and other public dog data at K = 4 ~ 8.

*LT: long-tailed DG, ST: Short-tailed DG, NT: no-tailed DG, J: Jindo, GW: Gray wolf, JRT: Jack Russell Terrier, AS: Australian Shepherd, ES: English Setter, BS: Brittany Spaniel, MS: Miniature Schnauzer, GR: Golden Retriever, R: Rottweiler, ESS: English Springer Spaniel, PWC: Pembroke Welsh Corgi

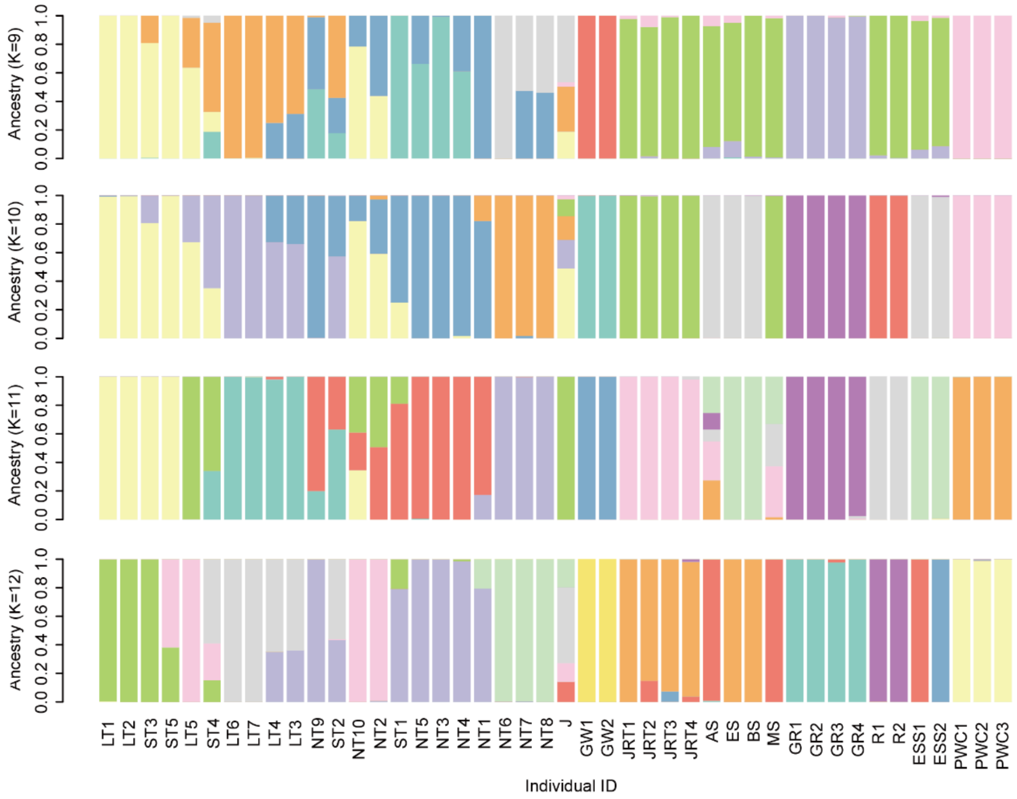

Supplementary S4-2 Fig. The population structure of DongGyeongi (DG) samples and other public dog data at K = 9 ~ 12.

*LT: long-tailed DG, ST: Short-tailed DG, NT: no-tailed DG, J: Jindo, GW: Gray wolf, JRT: Jack Russell Terrier, AS: Australian Shepherd, ES: English Setter, BS: Brittany Spaniel, MS: Miniature Schnauzer, GR: Golden Retriever, R: Rottweiler, ESS: English Springer Spaniel, PWC: Pembroke Welsh Corgi

Supplementary Table S1. Summary of re-sequencing of DongGyeongi

Sample	Species	Total reads	Alignment Rate	Coverage	Gap size	Depth
LT1	DongGyeongi	205,809,489	99.22%	99.32%	0.68%	16.48
LT2	DongGyeongi	175,600,370	98.62%	99.35%	0.65%	13.93
LT3	DongGyeongi	183,345,774	99.25%	99.34%	0.66%	14.62
LT4	DongGyeongi	201,722,990	99.24%	99.35%	0.65%	16.03
LT5	DongGyeongi	194,925,071	99.05%	99.29%	0.71%	15.53
LT6	DongGyeongi	182,905,364	99.16%	99.32%	0.68%	14.61
LT7	DongGyeongi	200,297,129	99.10%	99.33%	0.67%	16.14
NT1	DongGyeongi	191,946,935	99.20%	99.36%	0.64%	15.53
NT2	DongGyeongi	190,086,432	99.26%	99.35%	0.65%	15.38
NT3	DongGyeongi	195,203,967	99.26%	99.33%	0.67%	15.80
NT4	DongGyeongi	155,041,417	99.05%	99.31%	0.69%	12.53
NT5	DongGyeongi	191,246,094	99.25%	99.33%	0.67%	15.50
NT6	DongGyeongi	196,997,490	99.07%	99.32%	0.68%	15.91
NT7	DongGyeongi	203,102,582	99.09%	99.32%	0.68%	16.39
NT8	DongGyeongi	182,105,551	98.46%	99.41%	0.59%	14.66
NT9	DongGyeongi	187,748,148	98.53%	99.41%	0.59%	15.14
NT10	DongGyeongi	197,459,281	98.29%	99.41%	0.59%	15.82
ST1	DongGyeongi	166,480,263	98.52%	99.28%	0.72%	13.08
ST2	DongGyeongi	161,365,483	98.40%	99.26%	0.74%	12.58
ST3	DongGyeongi	156,137,329	98.53%	99.29%	0.71%	12.27
ST4	DongGyeongi	195,565,270	98.12%	99.41%	0.59%	15.55
ST5	DongGyeongi	202,575,531	98.36%	99.40%	0.60%	16.38
SRS1124460	Australian Shepherd	251,762,057	99.17%	99.43%	0.57%	20.24
SRS981383	Brittany Spaniel	50,322,431	99.36%	91.09%	8.91%	4.27
SRS1124453	English Setter	269,059,876	99.09%	99.39%	0.61%	21.60
SRS932160	English Springer Spaniel	301,544,915	99.07%	99.26%	0.74%	23.63
SRS1124454	English Springer Spaniel	312,121,416	99.07%	99.40%	0.60%	25.05
SRS981401	Golden Retreiver	369,233,985	99.28%	97.35%	2.65%	31.58
SRS932149	Golden Retriever	316,578,558	99.10%	99.34%	0.66%	25.16
SRS834021	Golden Retriever	223,860,814	99.25%	99.42%	0.58%	18.06
SRS1124457	Golden Retriever	306,093,842	99.15%	99.42%	0.58%	24.58
SRS932167	Jack Russell Terrier	285,944,083	98.88%	99.32%	0.68%	22.44
SRS932151	Jack Russell Terrier	422,185,312	99.25%	99.45%	0.55%	33.93
SRS932147	Jack Russell Terrier	298,368,533	99.17%	99.45%	0.55%	23.83
SRS1124459	Jack Russell Terrier	281,144,082	99.15%	99.43%	0.57%	22.55
DRS001112	Jindo	489,741,767	97.99%	99.22%	0.78%	39.00

SRS1124464	Miniature Schnauzer	55,638,696	98.60%	91.95%	8.05%	4.20
SRS732549	Pembroke Welsh Corgi	274,312,323	97.57%	99.12%	0.88%	20.66
SRS732550	Pembroke Welsh Corgi	249,274,578	98.18%	99.29%	0.71%	19.10
SRS732551	Pembroke Welsh Corgi	265,797,150	99.28%	99.33%	0.67%	21.46
SRS834058	Rottweiler	288,839,083	99.32%	99.39%	0.61%	23.41
SRS984795	Rottweiler	531,555,714	96.28%	99.08%	0.92%	43.36
SE05	Gray Wolf	153,327,608	98.85%	99.30%	0.70%	11.97
SE06	Gray Wolf	126,095,384	98.58%	99.23%	0.77%	9.93

52

Supplementary Table S2. Public data from NCBI

Name	SRA_Sample	SRA_Study	BioProject
Australian Shepherd	SRS1124460	SRP049358	PRJNA263947
Brittany Spaniel	SRS981383	SRP060516	PRJNA288568
English Setter	SRS1124453	SRP049358	PRJNA263947
English Springer Spaniel 1	SRS932160	SRP049358	PRJNA263947
English Springer Spaniel 2	SRS1124454	SRP049358	PRJNA263947
Golden Retriever 1	SRS981401	SRP060516	PRJNA288568
Golden Retriever 2	SRS932149	SRP049358	PRJNA263947
Golden Retriever 3	SRS834021	SRP049358	PRJNA263947
Golden Retriever 4	SRS1124457	SRP049358	PRJNA263947
Jack Russell Terrier 1	SRS932167	SRP049358	PRJNA263947
Jack Russell Terrier 2	SRS932151	SRP049358	PRJNA263947
Jack Russell Terrier 3	SRS932147	SRP049358	PRJNA263947
Jack Russell Terrier 4	SRS1124459	SRP049358	PRJNA263947
Jindo	DRS001112	DRP000492	PRJDB2266
Miniature Schnauzer	SRS1124464	SRP049358	PRJNA263947
Pembroke Welsh Corgi 1	SRS732549	SRP049358	PRJNA263947
Pembroke Welsh Corgi 2	SRS732550	SRP049358	PRJNA263947
Pembroke Welsh Corgi 3	SRS732551	SRP049358	PRJNA263947
Rottweiler 1	SRS834058	SRP049358	PRJNA263947
Rottweiler 2	SRS984795	SRP060516	PRJNA288568

Supplementary Table S3. Candidate selective sweep region discovered by three statistics including SweepFinder2's alpha, Tajima's D and Fst

Chrom	Start	End	Alpha	Tajima's D	Fst	Genes
5	64200000	64250000	0.00000132	-0.950583	0.221457	**ANKRD11**,
23	8200000	8250000	0.0000187	-1.05652	0.313067	EXOG,**ACVR2B**,
1	4000000	4050000	2.65E-05	-1.0547	0.247817	
1	14600000	14650000	1.05E-05	-0.663937	0.376311	KIAA1468,
1	47350000	47400000	5.00E-05	-1.514	0.230406	SNX9,
1	47400000	47450000	1.13E-05	-1.74477	0.278	SNX9,
1	50600000	50650000	2.96E-05	-1.11428	0.222921	
1	58900000	58950000	8.83E-06	-0.833723	0.390193	FAM184A,
1	58950000	59000000	6.98E-05	-0.959517	0.322326	FAM184A,
1	69050000	69100000	8.71E-06	-1.07598	0.280995	
1	75600000	75650000	1.03E-05	-1.79869	0.240039	KIF27,
1	113450000	113500000	5.54E-05	-0.855336	0.280921	
2	11650000	11700000	6.49E-05	-1.22626	0.273928	
2	33150000	33200000	8.76E-06	-1.36491	0.291481	
2	33200000	33250000	6.14E-06	-1.06467	0.386508	
2	33250000	33300000	6.03E-06	-0.764189	0.300027	
3	58200000	58250000	1.82E-05	-1.08923	0.275791	TBC1D2B,ADAMTS7,
4	27950000	28000000	1.30E-05	-0.777163	0.265029	
4	32550000	32600000	8.79E-06	-2.01731	0.255704	CCSER2,
4	32600000	32650000	4.88E-06	-2.33191	0.249818	CCSER2,
4	32650000	32700000	4.36E-06	-2.26212	0.244114	CCSER2,
4	32700000	32750000	8.47E-06	-1.52176	0.249508	CCSER2,
4	33600000	33650000	1.15E-05	-1.38786	0.305502	
4	44600000	44650000	6.42E-05	-0.798719	0.31933	
4	52100000	52150000	1.31E-05	-0.840401	0.294238	
5	41950000	42000000	1.25E-05	-0.685655	0.267027	RASD1,PEMT,MED9,
5	42100000	42150000	8.66E-06	-0.733995	0.284687	COPS3,NT5M,
5	56000000	56050000	3.00E-05	-0.62347	0.294587	ZYG11A,HES4,
5	60950000	61000000	2.26E-05	-0.98946	0.252189	CAMTA1,
5	64250000	64300000	1.40E-06	-2.19918	0.247671	ACSF3,CDH15,
7	56800000	56850000	1.17E-05	-0.770957	0.305415	CCDC178,
8	6150000	6200000	2.27E-05	-1.84056	0.433043	NOVA1,
8	6200000	6250000	2.59E-05	-1.57788	0.471663	NOVA1,
8	25400000	25450000	2.10E-05	-1.39217	0.33896	

8	61650000	61700000	5.13E-05	-1.20285	0.223288	TTC7B,
9	20200000	20250000	2.16E-05	-1.11966	0.273971	WNK4,BECN1,CCDC56,
9	27250000	27300000	1.97E-05	-1.12252	0.25483	
9	30450000	30500000	6.19E-05	-0.95301	0.374596	MMD,
9	33000000	33050000	5.10E-06	-1.70726	0.250509	RNF43,
9	33050000	33100000	2.16E-06	-2.09921	0.272245	HSF5,RNF43,
9	48050000	48100000	2.55E-05	-1.41929	0.271063	EHMT1,
9	48100000	48150000	1.06E-05	-1.43018	0.265985	EHMT1,
11	15450000	15500000	8.76E-06	-1.20242	0.371773	
11	31950000	32000000	1.07E-05	-1.09203	0.249003	
11	35500000	35550000	1.82E-05	-1.82156	0.277605	TTC39B,
11	48600000	48650000	3.93E-06	-1.76394	0.233789	
11	48650000	48700000	6.22E-06	-1.32811	0.428693	
11	48700000	48750000	1.74E-05	-1.18551	0.286047	
12	31100000	31150000	2.14E-05	-1.77276	0.618093	
12	38750000	38800000	1.82E-05	-0.916777	0.328335	
12	38800000	38850000	1.07E-05	-1.16297	0.314838	MEI4,
12	38850000	38900000	5.35E-05	-0.925428	0.36835	bacterial,MEI4,
12	44950000	45000000	8.11E-06	-2.34859	0.26444	
13	29200000	29250000	1.08E-05	-1.19488	0.246925	TMEM71,
13	35800000	35850000	1.55E-05	-0.974871	0.279205	GPR20,
13	35850000	35900000	9.39E-06	-1.60874	0.252363	MROH5,PTP4A3,
13	39600000	39650000	9.99E-06	-0.699909	0.251174	
13	44200000	44250000	1.49E-05	-1.2133	0.393441	SLAIN2,
16	5150000	5200000	1.55E-05	-0.989668	0.474452	
16	7450000	7500000	4.74E-06	-2.08424	0.369726	SSBP1,WEE2,
16	22350000	22400000	1.55E-05	-1.28488	0.243627	
16	24950000	25000000	2.70E-05	-0.865381	0.347113	
16	25500000	25550000	7.20E-06	-0.993095	0.538877	LOC475575,
16	37350000	37400000	7.94E-06	-1.28803	0.321979	
16	37400000	37450000	1.03E-05	-1.82147	0.359283	SGCZ,
18	10550000	10600000	1.23E-05	-1.93917	0.419215	LOC483249,
18	11350000	11400000	1.58E-05	-2.20933	0.287001	
19	1500000	1550000	1.13E-05	-0.881212	0.331855	
20	7400000	7450000	2.35E-05	-1.18957	0.388993	SLC6A11,
20	7450000	7500000	8.99E-06	-0.951969	0.250269	SLC6A11,
21	33200000	33250000	2.93E-06	-1.38475	0.338575	SBF2,
21	33250000	33300000	2.92E-06	-1.44485	0.254644	SBF2,

21	33300000	33350000	5.42E-06	-0.936234	0.282901	SBF2,
21	33350000	33400000	6.25E-06	-1.1653	0.263374	SBF2,LOC106560188,
22	4950000	5000000	4.00E-05	-1.67942	0.277712	LRRC63,
22	5000000	5050000	2.50E-05	-0.868865	0.239213	LCP1,
22	14300000	14350000	1.15E-05	-0.95389	0.231001	
22	15450000	15500000	6.36E-06	-1.08402	0.551344	
22	17500000	17550000	1.75E-05	-1.00336	0.520442	
22	17950000	18000000	2.88E-05	-1.15412	0.302921	
22	19800000	19850000	2.95E-05	-1.77066	0.45868	
23	40900000	40950000	3.95E-05	-1.34254	0.256633	
24	3900000	3950000	5.15E-05	-1.00925	0.285699	
25	16000000	16050000	9.81E-06	-1.76125	0.2893	
25	22650000	22700000	1.56E-05	-1.46481	0.389456	
25	22700000	22750000	1.56E-05	-1.9057	0.263161	
25	34950000	35000000	1.23E-05	-1.47059	0.334034	PIWIL2,
27	4050000	4100000	5.07E-06	-1.01866	0.226731	DIP2B,
27	12100000	12150000	1.10E-05	-0.927453	0.351448	
27	14700000	14750000	4.03E-06	-1.34297	0.366565	CPNE8,
27	42300000	42350000	5.99E-06	-2.03576	0.234656	SLC6A13,
27	42350000	42400000	5.93E-06	-2.16122	0.350995	KDM5A,
27	42400000	42450000	7.54E-06	-1.93598	0.331341	KDM5A,
29	4100000	4150000	5.54E-05	-0.660533	0.23354	
30	10100000	10150000	2.18E-05	-1.42084	0.244294	TGM5,CCNDBP1,TGM7,
30	11700000	11750000	1.31E-05	-1.71028	0.280795	GATM,
30	17250000	17300000	2.24E-05	-1.27524	0.288011	DMXL2,
30	19200000	19250000	1.14E-05	-1.39652	0.270791	WDR72,
31	5350000	5400000	2.06E-05	-1.48223	0.374229	
31	5400000	5450000	1.88E-05	-1.57673	0.418404	
31	12650000	12700000	1.46E-05	-0.845711	0.254599	USP25,
33	29800000	29850000	2.06E-05	-0.622624	0.359957	RAC1,
X	18800000	18850000	7.43E-06	-0.962282	0.333881	
X	18850000	18900000	5.79E-06	-1.36359	0.260553	
X	41550000	41600000	8.60E-06	-1.2404	0.347255	LOC480901,ZNF182,
X	63700000	63750000	6.19E-06	-1.48713	0.506906	
X	63750000	63800000	6.07E-06	-0.635592	0.402553	
X	78150000	78200000	5.35E-05	-0.887501	0.229911	
X	80750000	80800000	7.96E-07	-0.650903	0.492251	NUP62CL,PIH1D3,
X	81300000	81350000	1.31E-06	-1.82389	0.491915	

X	81350000	81400000	1.38E-06	-1.6765	0.46726	MID2,
X	81450000	81500000	1.43E-06	-1.66377	0.47723	TEX13B,LOC102157101,MID2,
X	81500000	81550000	1.46E-06	-2.03553	0.456104	LOC102157101,
X	81550000	81600000	1.51E-06	-1.74689	0.512779	VSIG1,
X	81600000	81650000	1.58E-06	-1.98044	0.346171	PSMD10,VSIG1,
X	81650000	81700000	1.64E-06	-1.89576	0.427664	COL4A6,ATG4A,
X	81700000	81750000	1.73E-06	-2.13179	0.43324	COL4A6,
X	81750000	81800000	1.83E-06	-1.60226	0.387646	COL4A6,
X	81800000	81850000	1.95E-06	-1.90443	0.234076	COL4A6,
X	86550000	86600000	0.0000456	-0.991426	0.268884	

Supplementary Table S4. No tail (NT)-specific non-synonymous SNVs

Chr.	Gene	Position	Long_tail							No_tail										Short_tail				
			LT1	LT2	LT3	LT4	LT5	LT6	LT7	NT1	NT1	NT3	NT4	NT5	NT6	NT7	NT8	NT9	NT10	ST1	ST2	ST3	ST4	ST5
1	T	54192143	C/C	C/C	C/C	C/C	C/C	C/C	C/C	C/G	C/G	C/G	C/G	C/G	C/G	C/G	C/G	C/G	C/G	C/C	C/C	C/C	C/C	C/C
1	ZNF329	99763301	C/C	C/C	C/C	C/C	C/C	C/C	C/C	T/T	C/T	C/T	C/T	C/T	T/T	T/T	T/T	C/T	C/T	T/T	C/C	C/C	C/T	C/C
11	ALDH7A1	15878370	G/G	G/G	G/G	G/G	G/G	G/G	G/G	A/A	G/A	G/A	G/A	G/A	A/A	A/A	A/A	G/A	G/A	G/G	G/G	G/G	G/A	G/G

Supplementary Table S5. Short tail (ST)-specific SNVs in CpG island and regulatory region

Type	Chr	Gene	Position	Long_tail							No_tail										Short_tail				
				LT1	LT2	LT3	LT4	LT5	LT6	LT7	NT1	NT1	NT3	NT4	NT5	NT6	NT7	NT8	NT9	NT10	ST1	ST2	ST3	ST4	ST5
**CpGi**	**15**	**SFRP2**	**51552556**	**G/G**	**G/G**	**G/G**	**G/G**	**G/G**	**G/G**	**G/G**	**G/G**	**G/G**	**G/A**	**G/A**	**G/G**	**G/G**	**G/G**	**G/G**	**G/G**	**G/G**	**G/A**	**G/A**	**G/A**	**G/A**	**G/A**
CpGi	15	ENSCAFG00000008160-TMEM154	50478004	G/G	G/G	G/G	G/G	G/G	G/G	G/G	G/G	G/G	G/G	G/A	G/A	G/G	G/G	G/G	G/G	G/G	G/A	G/A	G/A	G/A	G/A
CpGi	15	ENSCAFG00000008160-TMEM154	50477989	A/A	A/A	A/A	A/A	A/A	A/A	A/A	A/A	A/A	A/A	A/G	A/G	A/A	A/A	A/A	A/A	A/A	A/G	A/G	A/G	A/G	A/G
CpGi	1	ENSCAFG00000004420-PPM1N	110063692	T/T	T/T	T/T	T/T	T/T	T/T	T/T	T/T	T/T	T/T	T/T	T/T	T/T	T/T	T/T	T/G	T/G	T/G	T/G	T/G	T/G	T/G
Regulatory	2	PAPD5	65146096	G/G	G/G	G/G	G/G	G/G	G/G	G/G	G/G	G/G	G/G	G/G	G/G	G/G	G/G	G/G	G/G	G/G	G/A	G/A	G/A	G/A	G/A

Supplementary Table S6. Short tail (ST)-specific indels in CpG island and regulatory region

Type	Chr.	Gene	Position	Variant	Long_tail						No_tail											Short_tail				
					LT1	LT2	LT3	LT4	LT5	LT6	LT7	NT1	NT1	NT3	NT4	NT5	NT6	NT7	NT8	NT9	NT10	ST1	ST2	ST3	ST4	ST5
CpGi	22	GRTP1	60704992	C>CG	0/0	0/0	0/0	0/0	0/0	0/0	0/0	0/1	1/1	0/0	0/0	0/0	0/0	0/1	0/0	0/0	1/1	0/1	0/1	0/1	0/1	0/1
CpGi	23	U6	41668019	A>AC	0/0	0/0	0/0	0/0	0/0	0/0	0/0	0/0	0/0	1/1	0/0	1/1	0/0	0/1	0/0	1/1	1/1	1/1	0/1	1/1	0/1	1/1
CpGi	15	FHDC1	50815314	GCGGGGC>G	0/0	0/0	0/0	0/0	0/0	0/0	0/0	0/0	0/0	0/1	0/1	0/1	0/1	0/1	0/1	0/0	0/0	0/1	0/1	0/1	0/1	0/1
